# Supplementary material for: Fault zone heterogeneities explain depth-dependent pattern and evolution of slow earthquakes in Cascadia
Source: Nat Commun. 2021 Mar 30;12:1959. doi: 10.1038/s41467-021-22232-x (PMC8010077; doi:10.1038/s41467-021-22232-x)
Supplement: Supplementary file 1 — Supplementary Information [file 41467_2021_22232_MOESM1_ESM.pdf]

Supplementary Information for

Fault Zone Heterogeneities Explain Depth-dependent Pattern and Evolution of Slow Earthquakes in Cascadia

Yingdi Luo[1, 2]*, Zhen Liu [2]

Affiliations:
[1] JIFRESSE, University of California, Los Angeles, CA 90095
[2] Jet Propulsion Laboratory, California Institute of Technology, Pasadena, CA 91125

*Correspondence to: Yingdi Luo (luoyingd@jpl.nasa.gov)

Contents of this file

Supplementary Figures 1 to 7
Supplementary Table 1

This supplementary material includes Supplementary Figure 1 and 2 show additional observations of ETS propagation in Cascadia. Supplementary Figure 3 and 4 provide model settings of the uniform and bi-modular model. Supplementary Figure 5 provides additional snapshots of the results from uniform, bi-modular and linear models. Supplementary Figure 6 shows comparison of different asperity distributions of hexagonal, rectangular and random asperity distributions. Supplementary Figure 7 shows comparison of different tremor detecting threshold. Table 1 for detailed parametric settings of the numerical models presented in this study.

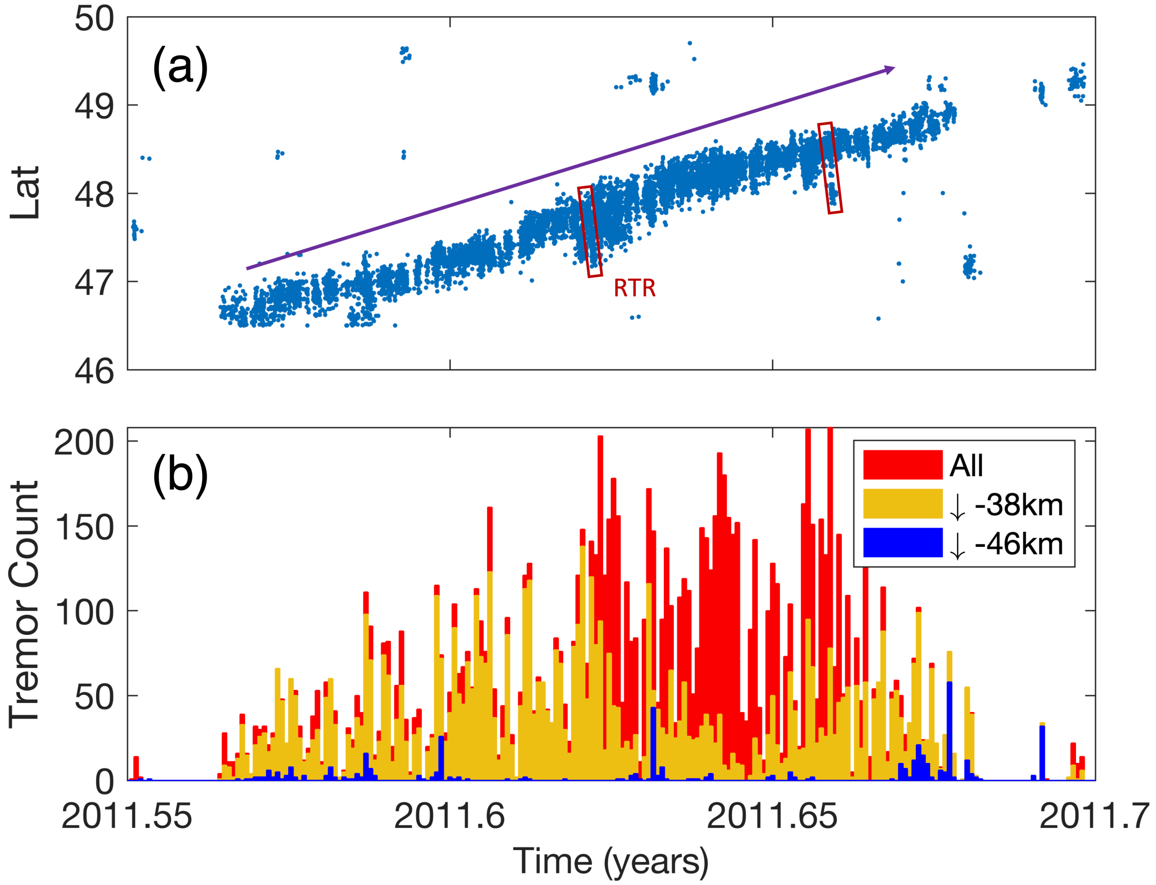

Supplementary Figure 1. An example of major episodic tremor and slow-slip (ETS) event that propagates unilaterally. Similar to Figure 2 in the main text. (a) shows the tremor catalog as a function of time and latitude. (b) the corresponding binned (6-hour) tremor activity as a function of time.

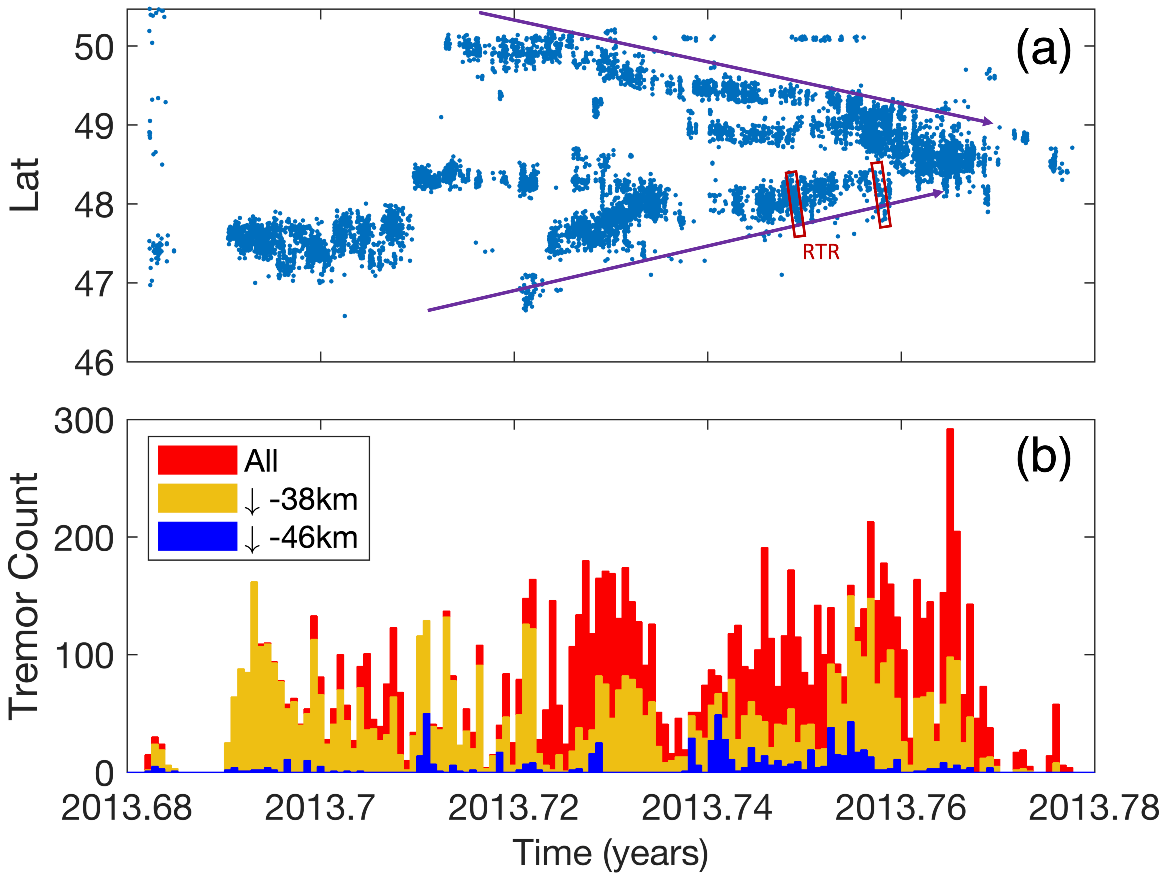

Supplementary Figure 2. An example of two major episodic tremor and slow-slip (ETS) events propagat towards each other and colliding. Similar to Figure 2 in the main text. (a) shows the tremor propagation as a function of time and latitude. (b) corresponding tremor activity binned in 6-hour window.

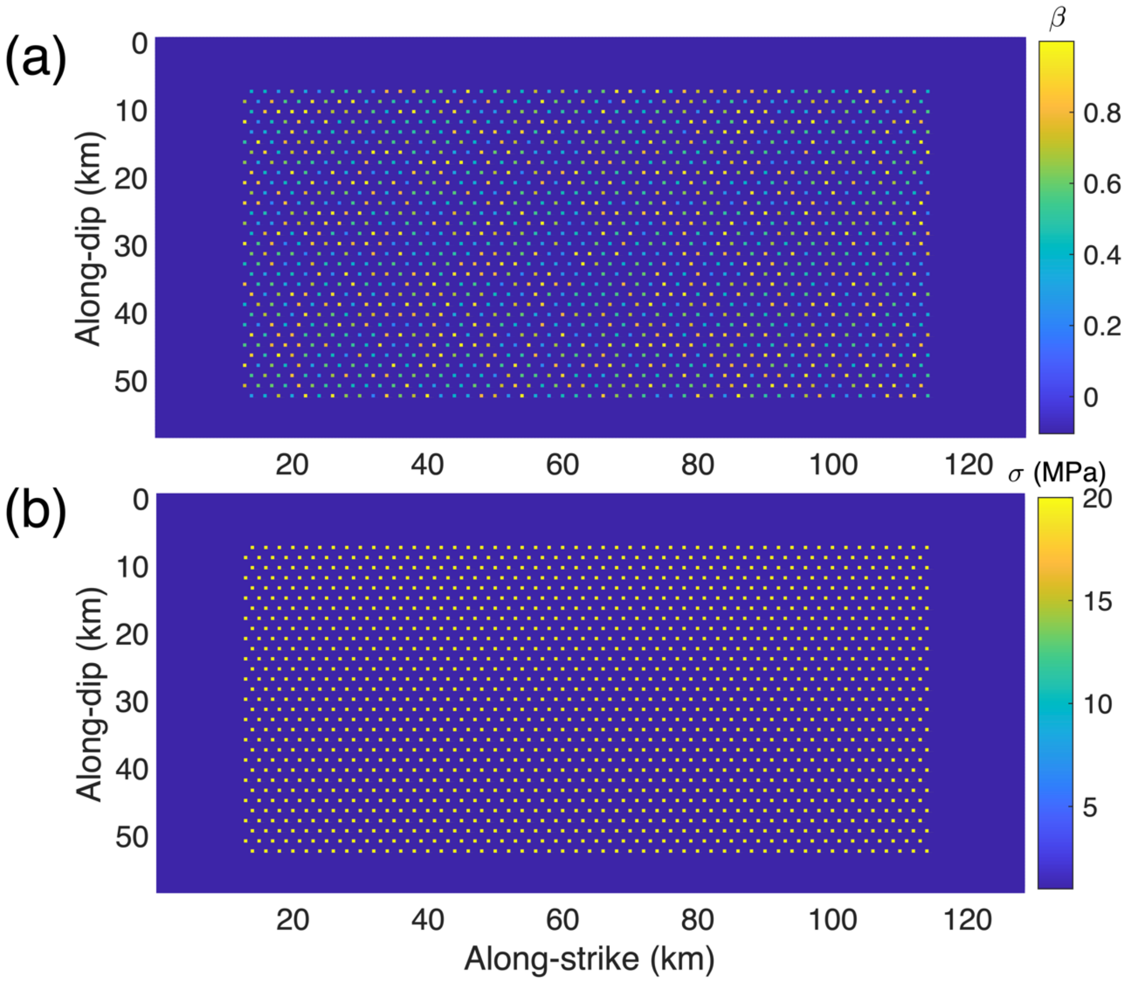

Supplementary Figure 3. Spatial distribution of frictional properties in the uniform model. (a) Fault-normal view of asperity criticalness $\beta = L_{asp}/L_c$(asperity size / critical length). (b) effective normal stress σ.

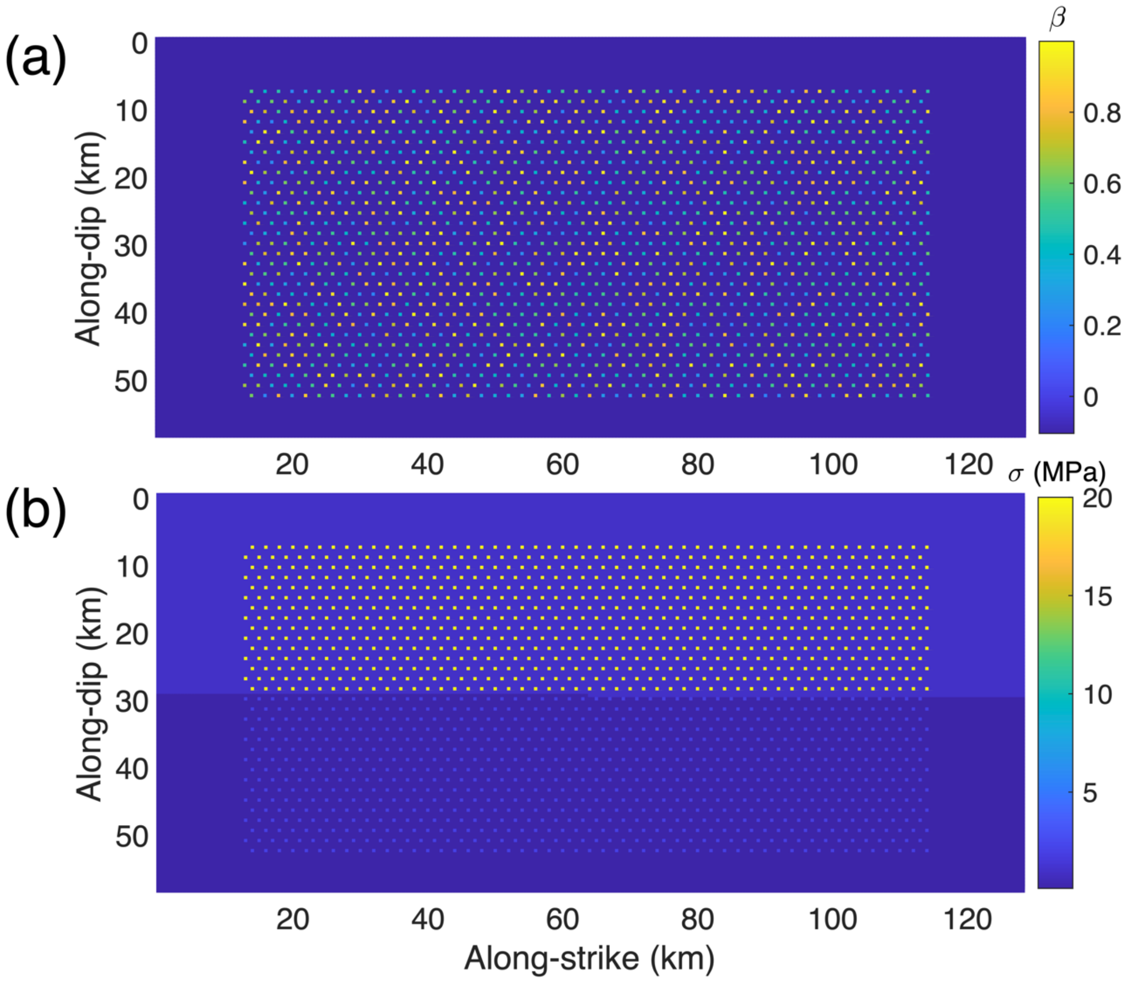

Supplementary Figure 4. Spatial distribution of frictional properties in the bi-modular model. (a) Fault-normal view of asperity criticalness $\beta = L_{asp}/L_c$. (asperity size / critical length). (b) effective normal stress σ.

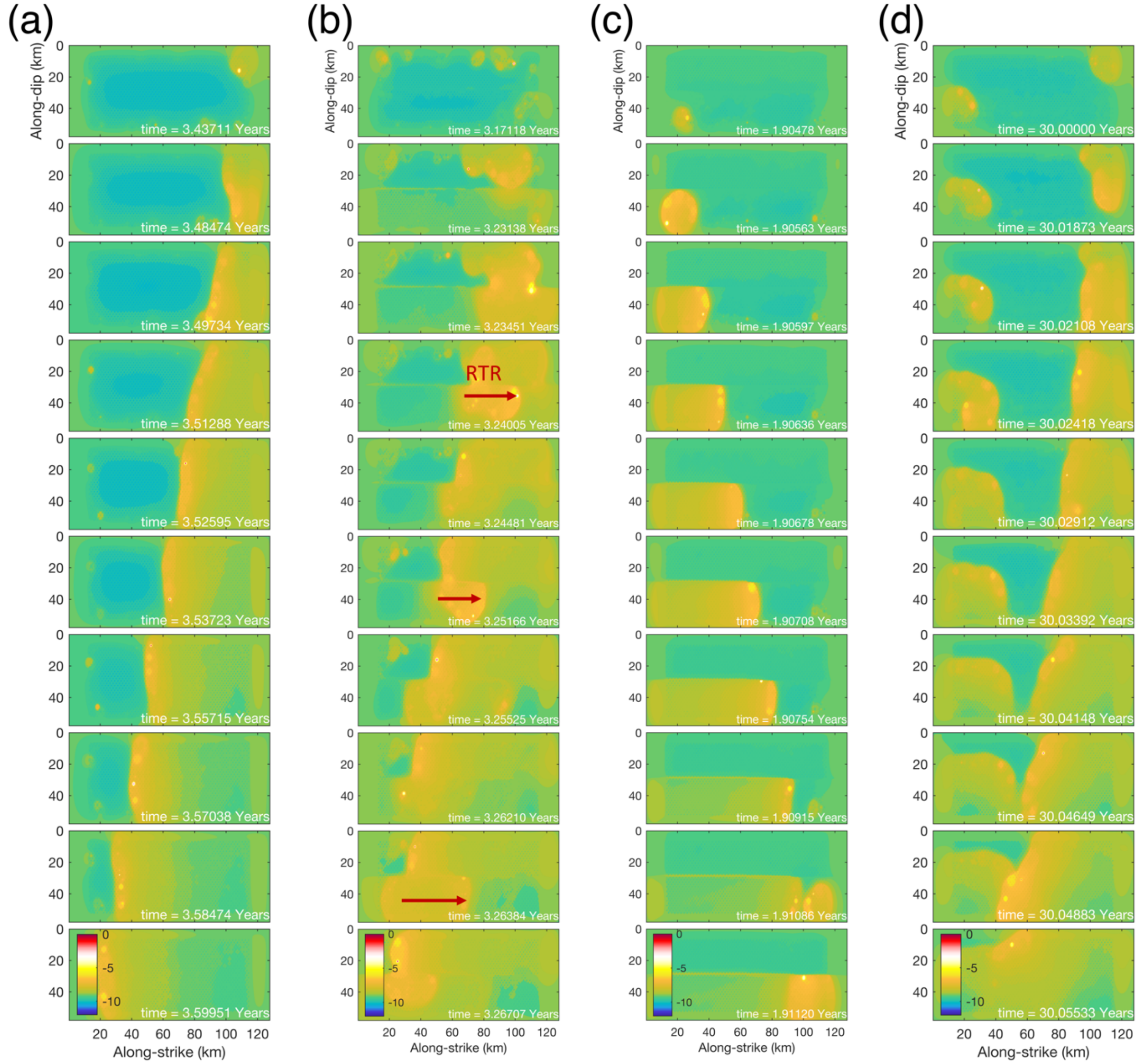

Supplementary Figure 5. Additional snapshots from model simulations. (a) A major ETS (episodic tremor and slow-slip) from the uniform model. (b) A major ETS from the bi-modular model. (c) A deep ETS from the bi-modular model. (d) A colliding major ETS from the linear model. Red arrows show plausible examples of rapid tremor reversals (RTRs).

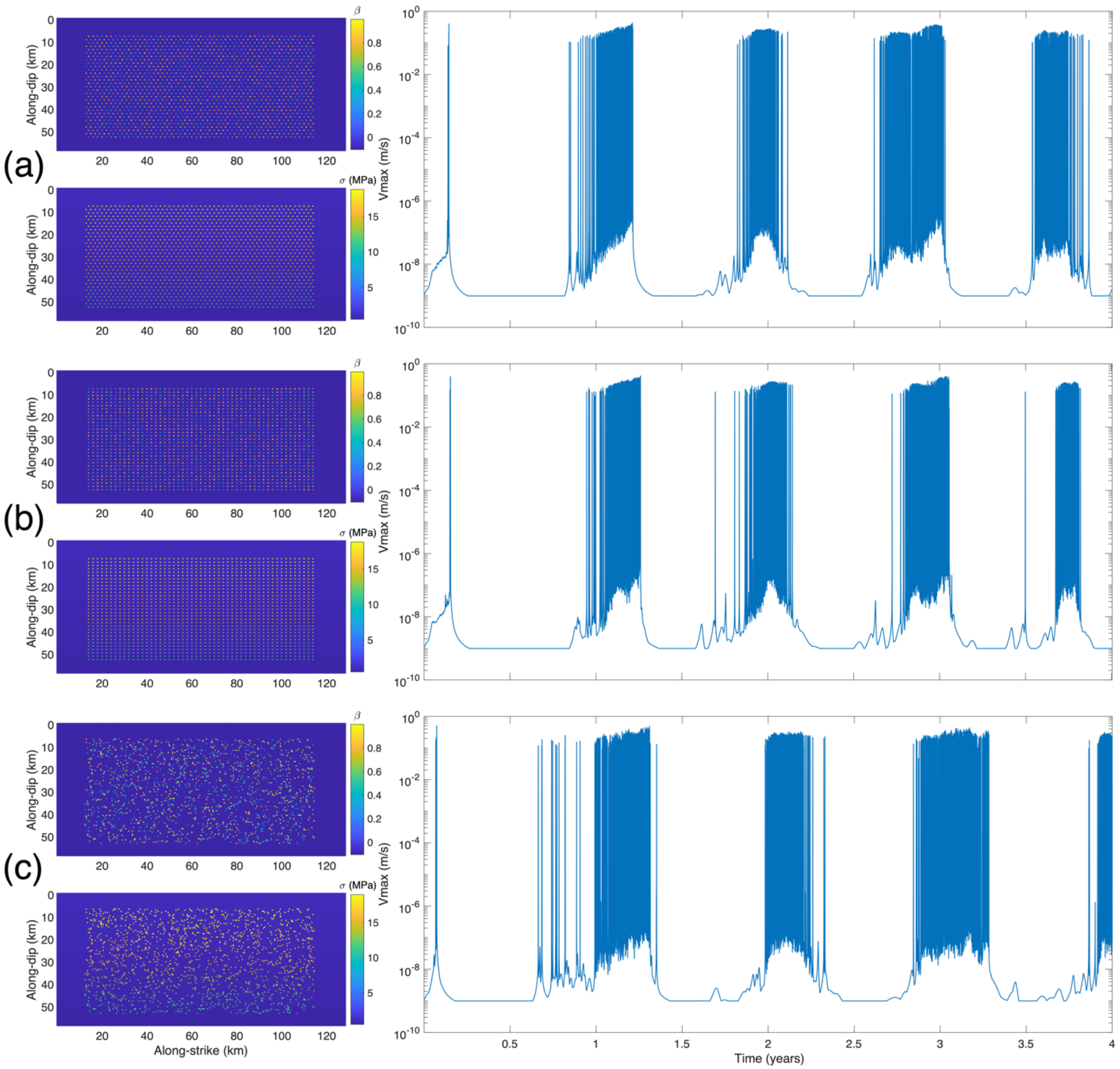

Supplementary Figure 6. Comparison of different asperity distributions. (a) hexagonal asperity distribution. (b) rectangular asperity distribution. (c) random asperity distribution. All the three models have same asperity criticalness (β) distribution (0.2-1.0) and Velocity-Weakening / Velocity-Strengthening (VW/VS) ratio (1/11). Left subplots: profile of asperity criticalness (β) and effective normal stress (σ). Right subplots: Global maximum slip rate as a function of time. The overall episodic tremor and slow-slip (ETS) patterns are very similar despite the actual spatial distribution of tremor asperities. Note to simplify the comparison the range of stress variation is 200%, instead of 1000% used in the Linear Model of main text, so only major ETS is reproduced.

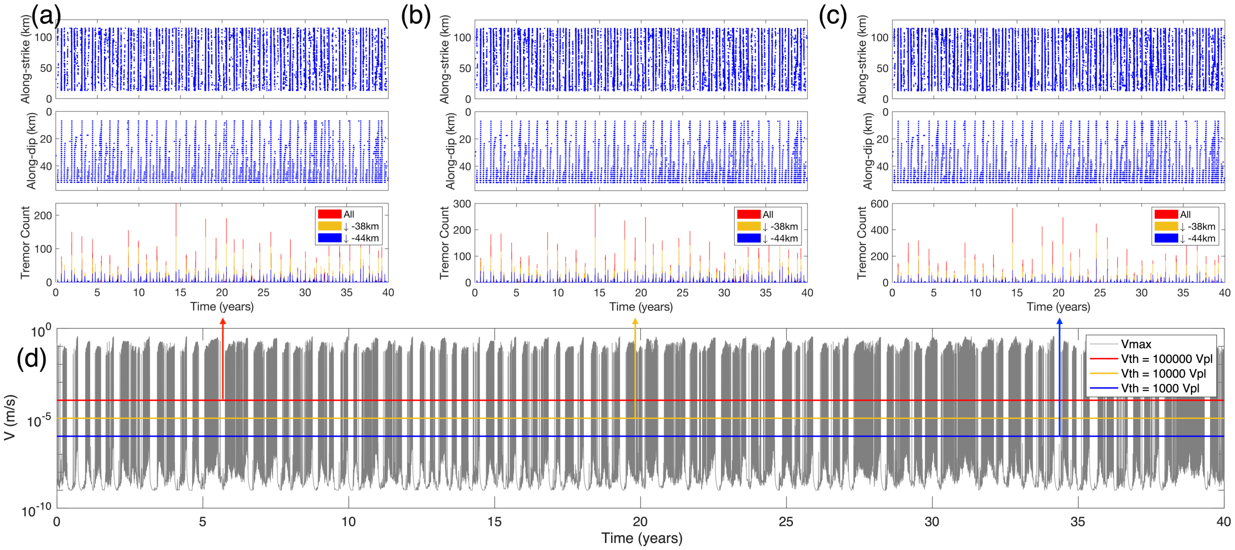

Supplementary Figure 7. Comparison of different tremor detecting threshold V_{th}: (a-c): Similar to Figure 5 in main text, showing detected tremor activities with strike- and dip-parallel projection, and tremor activities binned in 5-day window. (a) V_{th} = 100000 V_{pl} (tectonic loading rate); (b) V_{th} = 10000 V_{pl}; (c) V_{th} = 1000 V_{pl}; The overall ETS patterns remain the same despite V_{th} is varied by a factor of 100. (d) Global maximum slip rate as a function of time with three detecting thresholds used in (a-c) as solid red, yellow and blue lines.

Supplementary Table 1. Physical and numerical properties used in rate-and-state model

**physical properties**	**value**
fault length L	128 km
fault depth Z	30 to 50 km
fault dipping angel φ	20°
fault width W	58.5 km
shear modulus G	30 GPa
shear wave velocity V_s	$3000\ m \cdot s^{-1}$
reference friction coefficient μ^*	0.6
Tectonic loading rate V_{pl}	$10^{-9}\ m \cdot s^{-1}$
Asperity (VW) / background (VS) area ratio $f = A_{asp}/A_{bg}$	1/11
matrix characteristic slip distance Dc_{bg}	0.8 mm (uniform)<br>0.8 and 0.08 mm (bi-modular)<br>0.8 ~ 0.08 mm (linear)
matrix effective normal stress σ_{bg}	1 MPa (uniform)<br>1 and 0.1MPa (bi-modular)<br>1 ~ 0.1MPa (linear)
ratio of asperity / matrix effective normal stress $\alpha = \sigma_{asp}/\sigma_{bg}$	20/1
matrix constitutive parameter a_{bg} - direct effect	0.015
matrix constitutive parameter b_{bg} - indirect effect	0.01
asperity constitutive parameter a_{asp} - direct effect	0.005
asperity constitutive parameter b_{asp} - indirect effect	0.01
asperity criticalness $\beta = L_{asp}/L_c$	0.2~1.0
**Numerical properties**	**value**
cell size $dx = dw$	500m
solver accuracy	10^{-14}
tremor detecting threshold V_{th}	$10^4\ V_{pl}$ ($10^3\ V_{pl}$, $10^5\ V_{pl}$ verified)
