# Supplementary material for: Fault zone heterogeneities explain depth-dependent pattern and evolution of slow earthquakes in Cascadia
Source: Nat Commun. 2021 Mar 30;12:1959. doi: 10.1038/s41467-021-22232-x (PMC8010077; doi:10.1038/s41467-021-22232-x)
Supplement: Supplementary file 3 — Description of Additional Supplementary Files [file 41467_2021_22232_MOESM3_ESM.pdf]

Description of Additional Supplementary Files

File Name: Supplementary Movie 1
Description: (top) Movie of year 1~4 of slip rate snapshots from the uniform model. Color shows logarithmic slip rate ($m \cdot s^{-1}$). Blue: V < V_{pl} (locked); green: V ~ V_{pl} (creeping around plate loading rate); yellow: slow-slip; white-red: tremor (seismic, V > 10000 V_{pl}). (bottom) Time series of corresponding global maximum slip rate in logarithmic scale showing two major ETS episodes.

File Name: Supplementary Movie 2
Description: Similar to Movie 1 from the bi-modular model: 3 years slip rate history of two major ETS and about 15 deep ETS episodes.

File Name: Supplementary Movie 3
Description: Similar to Movie 1 from the linear model: 3 years slip rate history of a hierarchy of ETS with various sizes.

File Name: Supplementary Movie 4
Description: Similar to Movie 1 from the linear model but with more frequent outputs over 10 years (year 30 to 40).
